# A genetically explicit model of speciation by sensory drive within a continuous population in aquatic environments

**DOI:** 10.1186/1471-2148-7-99

**Published:** 2007-06-28

**Authors:** Masakado Kawata, Ayako Shoji, Shoji Kawamura, Ole Seehausen

**Affiliations:** 1 Department of Ecology and Evolutionary Biology, Graduate School of Sciences, Tohoku University, Sendai 980-8578, Japan; 2 Department of Integrated Biosciences, Graduate School of Frontier Sciences, The University of Tokyo, Kashiwanoha, Kashiwa, Chiba 277-8562, Japan; 3 Institute of Zoology, Department of Aquatic Ecology & Evolution, University of Bern, Baltzerstr. 6, CH-3012 Bern, and Center of Ecology, Evolution and Biogeochemistry, Swiss Institute for Environmental Sciences and Technology (EAWAG), CH-6047 Kastanienbaum, Switzerland

## Abstract

**Background:**

The sensory drive hypothesis predicts that divergent sensory adaptation in different habitats may lead to premating isolation upon secondary contact of populations. Speciation by sensory drive has traditionally been treated as a special case of speciation as a byproduct of adaptation to divergent environments in geographically isolated populations. However, if habitats are heterogeneous, local adaptation in the sensory systems may cause the emergence of reproductively isolated species from a single unstructured population. In polychromatic fishes, visual sensitivity might become adapted to local ambient light regimes and the sensitivity might influence female preferences for male nuptial color. In this paper, we investigate the possibility of speciation by sensory drive as a byproduct of divergent visual adaptation within a single initially unstructured population. We use models based on explicit genetic mechanisms for color vision and nuptial coloration.

**Results:**

We show that in simulations in which the adaptive evolution of visual pigments and color perception are explicitly modeled, sensory drive can promote speciation along a short selection gradient within a continuous habitat and population. We assumed that color perception evolves to adapt to the modal light environment that individuals experience and that females prefer to mate with males whose nuptial color they are most sensitive to. In our simulations color perception depends on the absorption spectra of an individual's visual pigments. Speciation occurred most frequently when the steepness of the environmental light gradient was intermediate and dispersal distance of offspring was relatively small. In addition, our results predict that mutations that cause large shifts in the wavelength of peak absorption promote speciation, whereas we did not observe speciation when peak absorption evolved by stepwise mutations with small effect.

**Conclusion:**

The results suggest that speciation can occur where environmental gradients create divergent selection on sensory modalities that are used in mate choice. Evidence for such gradients exists from several animal groups, and from freshwater and marine fishes in particular. The probability of speciation in a continuous population under such conditions may then critically depend on the genetic architecture of perceptual adaptation and female mate choice.

## Background

Reproductive isolation between populations may arise rapidly when male mating signals and female mating preferences are subject to divergent selection. The sensory drive hypothesis predicts that females mate more often with the male phenotypes that they detect more easily, and may evolve to prefer signals that are conspicuous and easy to detect in their environments [1,2]. Divergent sensory adaptation in different habitats could then lead to premating isolation upon secondary contact between populations. Speciation by sensory drive has traditionally been treated as a special case of speciation as a byproduct of adaptation to divergent environments in geographically isolated populations [1]. However, we were interested in exploring whether, if habitats are heterogeneous, local adaptation in the sensory systems may cause the emergence of reproductively isolated species even from a single unstructured population.

In many animals, visual signals, their environmental transduction, their perception, and the ambient light are important in courtship and other social interactions, as well as in predation. In guppies, orange spots are a visual cue in female mate choice and the conspicuousness of the spots can be affected by environmental light (e.g., [3-5]). Visual sensitivities of guppies might have evolved to detect food, which consequently led to a female preference for orange males as a byproduct [6]. In sticklebacks, male nuptial color varies with light environment [7,8]. Females prefer males with a larger area of red in environments where red is conspicuous [8], whereas red nuptial color is replaced by black in populations that nest in habitats where red is inconspicuous [7]. Such phenotypes can coexist as sympatric pairs of species where the environment is heterogeneous and both types of habitat exist [8]. In experiments with Lake Victoria cichlids, females of two closely related species preferred conspecific males in light environments where male color differences between species could be perceived, but showed no preferences where color variation could not be perceived [9]. In turbid waters, where color signals are week, males of Lake Victoria cichlids are far less brightly colored than in clear waters, and sympatric species often hybridize [10]. Within a species, females prefer males with more conspicuous bright nuptial coloration even under laboratory conditions where each male could easily be seen [11]. These observations indicate that females may actively prefer males with colors which the females perceive as intense or conspicuous.

Color perception is determined by several different components [12]. One important component is sensitivity at a given wavelength of light. Individuals with a given spectral sensitivity can detect lights of a certain range of wavelengths better or less well than individuals with other spectral sensitivities. In fish living in the optically dense medium water, the absorption spectra of visual pigments have been shown to correlate with spectral qualities of the environmental light. For instance, snappers (*Lutjanidae*) that live in the clear, blue water of outer shelf reefs have rhodopsins with sensitivity shifted to the blue end of the spectrum, while snappers that live in yellow-green inshore water have their sensitivity shifted to the green part of the spectrum [13]. Similarly, the retinas of cichlid fish inhabiting relatively blue-shifted environments are more sensitive to blue light than the retinas of those from red-shifted environments [14], and they differ in their behaviorally measured detection thresholds for blue and red light [15]. Similar observations have been made in sticklebacks [8]. Both positive and negative correlations between visual sensitivity at a given wavelength of light with the ambient abundance of that wavelength have been reported [16].

Closely related species of cichlid fish in Lake Victoria differ in their retinal absorption spectra [17]. Because the same species also differ in male breeding coloration, and because the most common breeding colors match the major peaks in the retinal absorption spectra, this led to the hypothesis that the evolution of the visual system might play a key role in the speciation process [10]. Spectral sensitivity is determined by visual pigments in the photoreceptor cells of the retina. Visual pigments consist of a chromophore and an opsin protein. In some species, environmental variation can induce differences in spectral sensitivity [18], but in other species such as sympatric species of cichlid fish, variation in sensitivity to light of different wavelengths correlates with heritable variation in the absorption spectra of opsins [14,15].

One mechanism of color vision tuning, is changing the amino acid sequence of opsin genes ('spectral tuning'; [19]), which causes changes in peak absorption spectra of the visual pigments. Fixed genetic differences were found at the long wavelength-sensitive (LWS) opsin locus between some closely related populations of Lake Victoria cichlid fish [20]. Terai et al. [20]suggested that interspecific variation in the amino acid sequence of opsin proteins might be related to variation in mate choice signals and food acquisition strategies.

Another way of changing color vision is by changing the relative amount of expression of different opsin genes. For instance, when a larger amount of LWS opsin is expressed in the retina than of medium and short wavelength-sensitive opsins (MWS and SWS), the individual could be more sensitive to light of longer wavelengths. Cichlids have 5 or 6 cone opsins but express only 3 of them as adults. Some species of Lake Malawi cichlids that inhabit different light environments express complimentary subsets of opsin genes [21]. When local environments differ such that they exert selection on visual sensitivity in different directions, evolutionary adaptation of the visual system by either of these mechanisms could lead to divergence between populations in female preference for male nuptial color. Upon secondary contact such divergence may cause premating isolation. The interaction between natural and sexual selection would have led to ecological by-product speciation [22,23].

In several groups of fishes with bright colors and mating preferences for bright colors, closely related species with different breeding color are commonly found in full sympatry, and have been suggested to have diverged at least partly in sympatry [8,24-31]. For instance, in rock-dwelling cichlids of African Lakes Victoria and Malawi, many pairs of closely related sympatric species differ in their nuptial coloration [30,32]. At the same time, the feeding ecologies and associated morphologies of the sympatric species are sometimes hardly differentiated [33]. Thus, the early stage of sympatric speciation in these cases could be driven by divergent sexual selection on male nuptial coloration.

Sympatric speciation had long been considered improbable from a theoretical point of view [34-39]. More recently several simulation models suggested that ecology-driven sympatric speciation can occur more easily than previously thought [40,41] (see review for [42]). Simultaneously, simulation models of sympatric speciation by sexual selection [43-45], and at least one mathematical model [46] have been proposed. Gavrilets [47] reexamined previous simulation models and developed mathematical models for sympatric speciation. He concluded that conditions for sympatric speciation are restrictive, and that some numerical simulations of resource competition-driven speciation [39,40] used unrealistic assumptions. Correspondingly, recent studies [48,49] that reexamined simulation models for sympatric speciation by sexual selection concluded that sympatric speciation is unlikely to occur unless initial genetic variation for female preference is large and symmetrically distributed.

The problem with most of the models for sympatric speciation though is that it is at best unknown and at worst unlikely that the assumptions and parameters used to generate disruptive selection and to allow for the emergence of stable polymorphisms in response to disruptive selection, are realistic [47,49,50]. Thus, the question how likely speciation through divergence of female mating preferences within a single population really is remains to be theoretically solved. Therefore, further development of the theory was needed to explain the sympatric occurrence of only weakly ecologically differentiated sister species with strongly divergent female mating preferences and male nuptial coloration [8,27,28,30,51-53]. Especially, it seemed important to investigate the likelihood of little ecologically differentiated speciation using more realistic models based on explicit genetic mechanisms of female choice and male secondary sexual traits.

In this paper, we investigate the possibility of speciation by sensory drive as a byproduct of divergent visual adaptation within a single initially unstructured population. We made the following assumptions: (1) Spectral sensitivity evolves as an adaptation to environmental (ambient) light regimes. (2) A female prefers to mate with a male the nuptial color of which reflects at the wavelength that she most intensely perceives. (3) The female's sensitivity to light of a given wavelength depends on the absorption spectra of her visual pigments. We then conducted individual-based simulations to examine the probability of speciation by sexual selection as a byproduct of local adaptation of the visual system in a heterogeneous light environment. By explicitly modeling the evolution of visual pigments and visual perception, we wish to examine if, and under which conditions, speciation within a continuous population can occur when no other causes of divergent selection are invoked.

## Results

### Outline of simulations

We conducted individual-based simulations in which the adaptive evolution of visual pigments and color perception were explicitly modeled to examine whether local adaptation and sensory drive can promote speciation along a short selection gradient within a continuous habitat and population. The simulated individuals are diploid and reproduce sexually. Males display nuptial colors, and females mate with a male and produce offspring (see the Life history section of Methods). The environmental light color in water varies with water depth (see the Habitat section of Methods). We assumed that individuals that can see light of the predominant ambient light color better have higher fitness. The ease with which an individual detects light of a given wavelength depends on the spectral sensitivity of its visual system (see the Spectral sensitivity and visual perception section of Methods). Individual variation in spectral sensitivity was assumed to be controlled by 3 opsin genes with different absorption spectra and the relative extent of their expression. We modeled the mechanism by which an individual with a given set of opsin genes perceives male nuptial colors and predominant environmental lights. Individual fitness is determined by local population density and an individual's sensitivity to the predominant light color in the local environment (see the Environment-dependent female fitnesssection of Methods). Male nuptial color is under polygenic control; spectral sensitivity is controlled by allelic variation at the opsin genes and polygenes that control the relative extent of expression of the opsin genes (see the Genetic control and variationsection of Methods). Mutations in the opsin genes can cause changes in the absorption spectrum and hence, spectral sensitivity (see the Mutation model section of Methods). Females search for mates within their mating area, and they actively prefer males whose nuptial color reflects at wavelengths that they can detect most easily (see *Mating and female mating preference*). Females produce offspring depending on their fitness, and the offspring disperse from their birth sites (see *Dispersal and neighborhood size*). The simulation procedures used have been reported in *Simulation sequence*, and the parameters used have been reported in *Parameters examined *and Table 1.

**Table 1:** Symbols and definitions in the model with values of parameters.

Sympol	Definition	Value
x, y	*xy *coordinates location within the habitat	x = 1000–5000, y = 1000
I(λ)	The ambient illumination spectrum	-
S_m_(λ)	The reflectance spectrum of the male nuptial color	-
S_e_(λ)	The predominant ambient light spectrum	-
R_i_(λ)	The absorption properties of the photoreceptor i	-
P(S)	The total sensitivity for an object with reflectance spectrum (S)	-
q_i_	The quantum catch of photoreceptor i adapted to its background	-
k_R_	The relative contributions to overall sensitivity by LWS cone opsin	-
k_G_	The relative contributions to overall sensitivity by MWS cone opsin	-
k_B_	The relative contributions to overall sensitivity by SWS cone opsin	-
λ_R1_, λ_R2_	The peak absorption wavelengths of allele 1 and 2 of LWS opsin gene	-
λ_G1_, λ_G2_	The peak absorption wavelengths of allele 1 and 2 of MWS opsin gene	-
λ_B1_, λ_B2_	The peak absorption wavelengths of allele 1 and 2 of SWS opsin gene	-
E_c_	The mean (peak) of the distribution of I(λ)	-
σ_E_	The width (standard deviation) of the distribution of I(λ)	6–16
σ_m_	The width (standard deviation) of the reflectance spectrum of male coloration	6–16
G_E_	The steepness of the gradient in predominant light color (peak wavelength)	0–0.225
G_s_	The steepness with which the width of I(λ) decreases with depth	0.025
G_I_	The steepness with which light intensity decreases with depth	0.0075
E_I_	Light intensity	-
r	Reproductive rate of an individual	1.6
N	The number of individuals within home range (radius of home range = 50)	-
E_max_	Maximum perception above which perception intensity cannot increase	22
K	Carrying capacity within the home range of an individual	5–15
V	Strength of selectioin	100
F(S)	The strength of female preference for males with reflectance spectrum S_m_	-
M	A female's mating area	75–300
μ	Mutation rates per generation	10^-5^,10^-3^,10^-4^, 10^-6^, 10^-7^, 10^-8^
d	The standard deviation of a normal distribution in which random dispersal distances were generated.	50–225
α	The strength of female preference relative to perception intensity	1–14
f	The cost of mate preference: the strength of female preference for the preferred male is reduced by f	-
c	The coefficient of the cost for mating preference	0–0.00005

### Observed speciation

**Figure 1:**
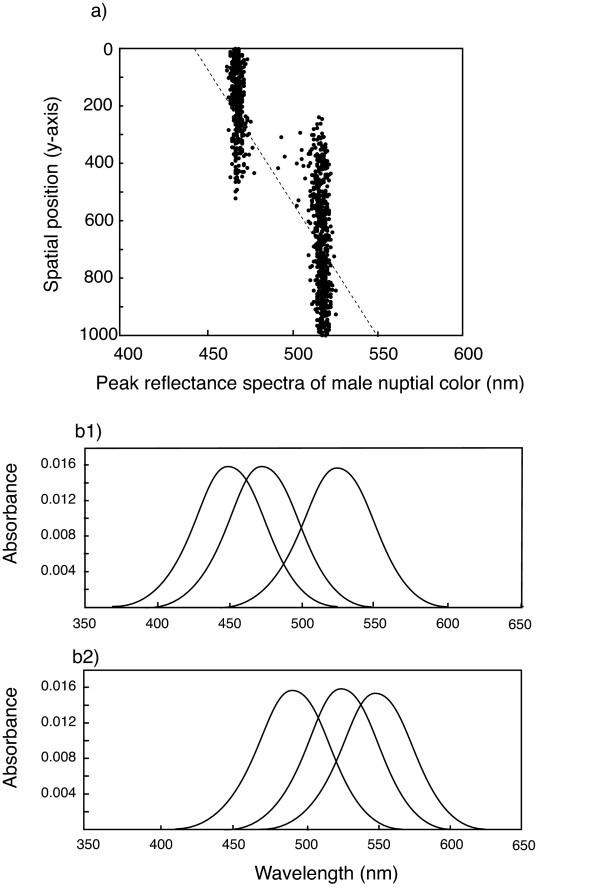
**Spatial distribution of individuals and the reflectance spectra of male nuptial colors after speciation occurred (a) and the absorption spectra of three visual pigments (SWS, MWS and LWS) for two individuals which are reproductively isolated (b1 and b2)**. Broken line indicates environmental wavelength to which individuals can adapt.

Speciation within 5000 generations occurred within a large range of parameter combinations. When it happened, male nuptial color diverged into two different colors (e.g., blue and red) and there were no or only a few males remaining with intermediate color (Fig. 1a). The absorption spectra of one or two opsin genes shifted towards blue or red generating two groups of females preferring to mate with males of either one of the two different colors (Fig. 1b). Thus, a population in which females preferred males with the peak reflectance of their nuptial coloration at short wavelengths became reproductively isolated from a population in which females preferred males with the peak reflectance of their nuptial coloration at long wavelengths. Upon completion of reproductive isolation, the depth distributions of the two groups were adjacent with large overlap (Fig. 1a). In rare cases, speciation resulted in three species with three different male nuptial colors, female preference classes and adjacent but overlapping depth distributions.

### Effects of environmental gradient, dispersal distance and mating area on speciation

**Figure 2:**
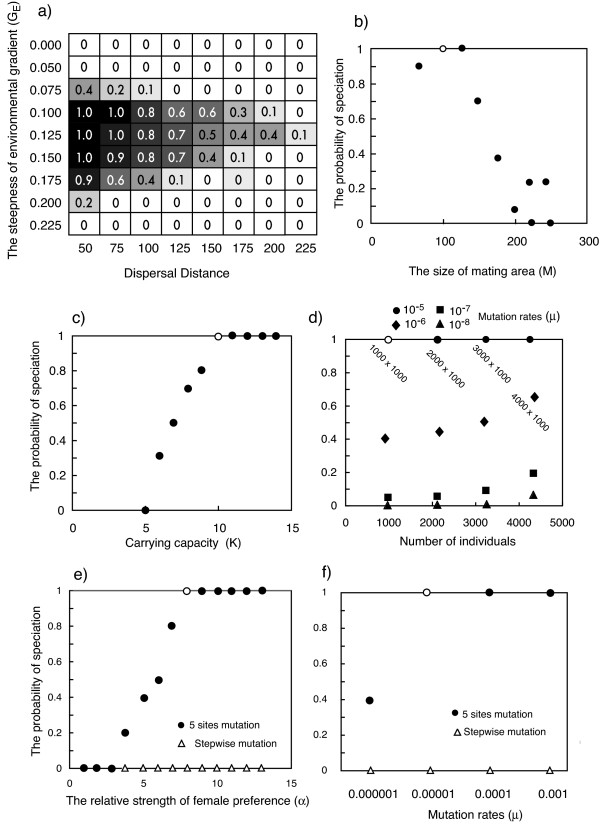
**The effects of environmental gradient and dispersal distance (a) and the effect of the size of mating area (b), carrying capacity (c), the size (horizontal extension) of habitat [x axis] (d), the relative strength of female preference (e), mutation mode, and mutation rate (f) on probability of speciation on probabilities of speciation**. G = 0.10 and d = 50 except (a), M = 100 except (b), K = 10 except (c), habitat size = 1000 × 1000 except (d), α = 8 except (e), μ = 0.00001 except and 5 sites mutation model except (d and f), Male nuptial color is controlled by 100 loci. a) M = 100; b) d = 50, G = 0.10, Each cell in (a) and each value in (b) is a mean of 10 replicate simulations. Parameter values used as background in exploration of individual parameter space are highlighted (open circle).

For exploration of parameter space permissive of speciation, we modified each parameter, starting from a combination of parameters in which speciation was frequently observed (see Table 1 for the definitions of parameters): habitat size (x axis) = 1000, K (carrying capacity) = 10, d (the standard deviation of dispersal distance) = 50, M (mating area) = 100, G_E _(the steepness of the gradient in predominant light color) = 0.10, μ (mutation rates for all loci = 0.00001, 5 sites mutation model for opsin genes (see *Mutation model *senction in Methods), k (the relative contribution by an opsin gene to overall sensitivity, e.g. through differential expression of opsin genes) controlled by 10 additive loci, α (the strength of female preference relative to perception intensity) = 8, male nuptial color controlled by 100 additive loci. Fig. 2a reports the probabilities of speciation for different combinations of the steepness of the environmental gradient of predominant light color (ambient peak wavelength), (G_E_) and the dispersal distance of offspring (d). Speciation occurred most frequently when dispersal distance was small (standard deviation of dispersal distance 5–15% of the length of the environmental gradient) and G_E _was intermediate (G_E _= 0.10–0.15). Similar to dispersal distance, increasing the mating area (M) decreased the probability of speciation although the speciation probability for the smallest mating area modeled (M = 75 = 7.5% of the length of the environmental gradient) was smaller than those for M = 100 and 125 (Fig. 2b). With mating areas of a diameter of 22.5% and more of the length of the environmental gradient, no speciation was observed. Similarly, speciation became rare when the standard deviation of dispersal distance exceeded 22.5% of the length of the environmental gradient (Fig. 2a). Hence, within the above detailed parameter set, frequent speciation required that standard deviation of dispersal distance and diameter of the mating area do not exceed 22% of the length of the environmental gradient.

### Effects of carrying capacity and habitat size on speciation

Increasing carrying capacity (K) or habitat size (along the selectively neutral x axis) and hence population size, increased speciation rates considerably (Fig. 2c and 2d). Varying carrying capacity (hence population size and density simultaneously) had larger effects than varying habitat size (hence population size alone). Thus, even with low mutation rates (10^-8^) in opsin genes causing changes in absorbance spectra, speciation occurred when the habitat size was large (Fig. 2d). Decreasing mutation rates reduced the probability of speciation (Fig. 2d), but the relative strength of female preference (α) also affected it (Fig. 2e). With the "five sites mutation" mode for opsin gene evolution, the probability of speciation increased with increasing mutation rate (μ)(Fig. 2f). When the mutation mode was "step-wise" (see *Mutation model *section of Methods), speciation never occurred even with high mutation rates.

### Effects of genetic control and spectral width of male nuptial color on speciation

**Figure 3:**
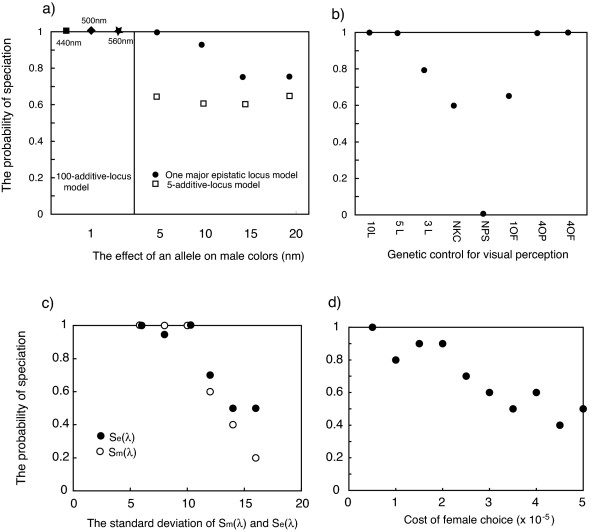
**The effects of genetic control of male nuptial color (a), different forms of genetic control of opsin gene differential expression and the number of opsin genes (b), the width of reflectance spectra of male color (S_m_(λ)) and predominant environmental color (S_e_(λ)) (c), and the cost of female choice (d) on the probability of speciation**. Habitat size = 1000 × 1000, K = 10, α = 8, μ = 0.00001, M = 100, d = 50, G = 0.10, 5 sites mutation model. Filled square, male nuptial colors controlled by 100 loci (*100-additive-locus model*), initial nuptial color and peak perception intensity 440 nm; Star, male nuptial colors controlled by 100 loci (*100-additive-locus model*),, initial nuptial color and peak perception intensity 560 nm; filled diamond, male nuptial colors controlled by 100 loci (*100-additive-locus model*), initial nuptial color and peak perception intensity 500 nm. Open squares, male nuptial color controlled by 5 loci (*5-additive-locus model*); filled circles, male nuptial color controlled by 4 additive loci and one major locus (*one major epistatic locus model*). Male nuptial color was controlled using 100 loci (*100-additive-locus model*) for b, c, and d. 3L, 5L, and 10L, k (the relative strength of electric signal and/or relative amounts of the three opsin gene expression) controlled by 3, 5 and 10 loci, respectively (*3-, 5- and 10-locus model*); NCK, *No differential expression and no electric signal change model*; NPS, *No peak shift model*; 1OF, *One locus on-off switch model*; 4OP, 4 opsin genes model; 4OF, *4 opsin genes model with one locus on-off switch model*.

The probability of speciation was similar when male nuptial color was controlled by 100 loci with additive effects of 1 nm and when it was controlled by one major epistatic locus plus additive effects of 5 nm or 10 nm at four loci (see *Male nuptial coloration *section of Methods). The probability in both models was somewhat higher than when male nuptial color was controlled by a major recessive locus (results not shown), or only by 5 additive loci (Fig. 3a). The probability of speciation was higher when the phenotypic effect of individual alleles at the additive loci was 5 or 10 nm, than when individual alleles had a larger effect (Fig 3a).

The width of the reflectance spectra of male nuptial coloration did not affect the results when it was about 60 nm (σ_e _= 10) or narrower. Above 60 nm spectral width, the probability of speciation decreases with increasing width. However, speciation occurred quite frequently even when the reflectance spectrum was 100 nm (σ_e _= 16) wide (Fig. 3c). We find that the relationship between the probability of speciation and the width of the ambient light spectrum (σ_e_) is similar to the relationship with the width of the reflectance spectra of male nuptial coloration. Speciation is frequently observed even with an ambient light spectral width of 100 nm.

### Effects of genetic control of visual sensitivity on speciation

The probability of speciation did not differ much among models with different numbers of loci controlling differential opsin gene expression (see *Genetic control and variation of visual perception *section of Methods)(Fig. 3b). However, in the model with completely fixed expression, speciation probabilities were lower than in others. The ''no peak shift model'', in which the wavelength of peak absorbance of individual opsin genes could not evolve, but differential expression could, was not permissive of speciation. The 4 opsin genes model yielded the same probabilities of speciation as the 3 opsin genes models (Fig. 3b).

When the minimum distances between the absorption peaks of LWS, MWS and SWS opsin proteins were constrained to 10 nm, the results were not different to those of the unconstrained model. When the minimum distances were constrained to 20 nm, the probability of speciation decreased slightly from 1.0 (unconstrained) to 0.8 in parameter combination of G = 0.125 and Dispersal distance = 50 in Fig. 2a. However, if we changed α (relative strength of female preference) from 8 to 10, the probability of speciation did not differ between constrained and unconstrained models of spectral tuning.

### Effects of the cost for female choice on speciaton

Increasing the cost for female choice reduced the probability of speciation (Fig. 3d, see *The cost of female choice *of Methods section). However, even if the cost of choice for the most distant males was 0.0002 (i.e, when the distance between female and male was 100 (= 10% of the length of the habitat along the y-axis), the strength of the preference decreased by 20%), the probability of speciation was not much different from simulations without costs.

### No effects of starting conditions

Importantly, the probability of speciation was not different whether initial male nuptial color and perception sensitivity were intermediate or shifted to long or short-wave color (Fig 3a).

## Discussion

### Speciation outcomes and their mechanisms

Several modeling studies have suggested that sympatric speciation through divergence of female mating preferences and associated male ornaments is theoretically possible, but it is unknown whether the assumptions and parameters used for these models are realistic. In our model, the evolution of visual pigments and color perception in a heterogeneous environment were explicitly modeled using an environmental gradient of realistic length and steepness. In this model sexual selection can, through sensory drive, promote speciation within a continuous and initially unstructured population as a result of local adaptation of the sensory system. When the light environment was spatially homogeneous or if adaptation of the visual system to local light environments was not possible, speciation never occurred in our models (results not shown). In addition, relatively small to moderate dispersal distance (standard deviation of dispersal distance < 22% of the length of the environmental gradient) of offspring was required for speciation. Hence, speciation within a single population may occur when environmental heterogeneity requires divergent local adaptation in a sensory modality that is used in mate choice, a situation that may not be uncommon in nature [1].

Speciation in our model is parapatric by theoretical definition [50], but the short environmental gradient and the weak spatial population structure that is sufficient for speciation (standard deviation of dispersal distance up to 22% of the length of the environmental gradient), reflected by 25–40% spatial overlap after speciation, make our model entirely applicable to most cases of speciation referred to as "sympatric" in the empirical literature. These include cichlid fish species flocks in Cameroonian and Nicaraguan crater lakes, the red and blue sister species of Lake Victoria cichlids, e.g. in the genus *Pundamilia*, sympatric pairs of limnetic and benthic chars, whitefish and sticklebacks in postglacial lakes, and sympatric incipient species of coral reef fish such as hamlets. In most of these cases, sympatric species differ slightly in the modal depths of their breeding sites along sloping lake bottoms [54-57], or differ otherwise in the background color of breeding sites [8]. Mating areas and dispersal ranges of ≤ 22% of the environmental gradient (e.g. the length of the shore slope from the shallowest part of the species' depth range of the shallow-dwelling species to the deepest part of the spawning depth range of the deeper-dwelling species) are in these cases not unrealistic.

The mechanism of speciation in a spatially distributed population experiencing spatially heterogeneous stabilizing selection with a linearly varying optimum has been controversial. Doebeli and Dieckmann [58] argued that ecological competition causes branching of populations, but Gavrilets [47] questioned this interpretation. Bridle, Butlin, and Kawata (submitted) simulated a spatially heterogeneous habitat with a linearly varying optimum without assuming mate choice. Migration loads (i.e., reduced fitness because of unfit migrants) were the highest at the center of the habitat since the populations at the center receive migrants from both directions along the ecological gradients. Thus, such migration loads might lead to disruptive selection and simultaneously reduced mating success of the intermediate phenotype that in turn might have caused speciation in our sensory drive model.

Boundary effects may cause the separation of 2 groups when there is no immigration from the outside into the habitat [47]. Speciation is more likely to occur at the edges of the habitats since the peripheral demes are subject to one-sided immigration, which distances them from neighboring demes. With regard to our results, however, boundary effects were unlikely to play a role in speciation since the spatial distribution of the emerging species was such that they could not have arisen from boundary effects [47].

### Empirical support for the assumptions of the model

We assumed that individuals that can see the predominant spectrum of the ambient light better have higher fitness. Both positive and negative correlations between visual sensitivity at a given wavelength of light and the abundance of that wavelength in the ambient light have been reported [8,13,15,16,59]. We assumed that the visual system evolves such that peak sensitivity tracks the ambient light spectrum [13,15]. This assumption is reasonable even if evolutionary adaptation of visual systems maximizes contrast vision and background matching simultaneously, as in trichromatic fish [60], because in such cases typically the extreme visual proteins are selected to track the shoulders of the mode in the ambient spectrum.

In our model, we assume that females prefer to mate with males whose nuptial coloration they can see better, and that differences in mating preferences among females depend on differences in their perception of colors. This might be a reasonable assumption since several studies [8,10] suggested that females of sexually dichromatic fish tend to prefer to mate with males, the nuptial colors of which they perceive most intensely. Studies on sympatric Lake Victoria cichlids showed that sister species that differ in their female mating preferences for male nuptial coloration [9], also differ in the peak absorption spectra of their opsin genes [14], and differ indeed in their visual sensitivity to red and blue light [15]. Our model may hence well apply to these sympatric species.

We assumed that the causes of individual variation in spectral sensitivity involve variation in the absorption spectra of visual pigments (opsins) and the expression patterns of different opsin genes in the photoreceptor. Endler et al. [61] showed that light sensitivity changed in response to artificial selection in guppies. Recent studies on cone visual pigments of Lake Malawi cichlids [21] and zebra fish [62] indicated that the relative expression levels of different opsin genes reflect spectral sensitivity of the fish. Also for Lake Victoria cichlids of the genus *Pundamilia*, variation in the amounts of expression of three cone opsin genes [14] is related to variation in color vision [14] and the differences in expression between species might have a genetic basis [14]. Thus, in the present model, individual variation in spectral sensitivity was assumed to be controlled by absorption spectra of three opsin genes (short, medium and long wave sensitive opsin genes, SWS, MWS and LWS) and the relative amount of expression of these genes.

We simulated the evolution of three photoreceptors expressing three different opsins to model the system of Lake Victoria cichlid [14]. In many perch-like fishes, such as cichlids and many coral reef fishes, there are five or six different opsin genes, but often only three are expressed in the cones [14,21]. Many fishes have more than three opsin genes. However, we did some simulations with four opsin genes too, and observed similar or slightly higher speciation probabilities. A larger number of opsin genes might facilitate speciation because the probability of occurrence of mutations that shift peak absorption spectra is higher.

The mechanisms of genetic control of female mating preferences are poorly understood. Mate preferences of female guppies might be controlled by several genes [63-66], and that of female Lake Victoria cichlid fish perhaps by more than one gene too [67]. In our model, we assume that variation among females in their perception of light of different wavelengths translates into variation in mating preferences. Several studies [8-10,68] suggested that females tend to mate with those males the nuptial colors of which are most intensely perceived by them. Maan et al. [15] showed that in *Pundamilia*, females of a species with blue male nuptial coloration detected light of shorter wavelengths (blue) more easily than females of a species with red male nuptial coloration, and vice versa. This perception difference coincides with differences in absorption spectra of LWS opsin alleles and differences in the relative amounts of expression of different opsin genes [14]. Thus, our assumptions may not be unrealistic.

In our model, female's relative liking for male color of a given wavelength increased nonlinearly with increasing visual sensitivity to male color. With increasing α (the strength of female preference relative to perception intensity), females are more likely to mate with males with the nuptial color that is most intensely perceived. This assumption might be supported by psychophysical experiments on the relationship between visual perception and decisions. Assume that animals choose between two objects according to their relative perception of these objects. The probability of choosing the more strongly perceived object increases nonlinearly with increasing difference in perceptual sensitivity to the two objects (e.g.,[69]). Thus, it is reasonably assumed that if a female encounters two males with different nuptial colors, she tends to choose the male perceived as more bright with higher probability than expected from the difference in perception intensity.

Some of the assumptions that we made to model visual perception and female preference may be overly simplistic. First, it is generally unknown how opsin gene expression is genetically controlled. In the bluefin killifish, there is both heritable and environmental variation in opsin gene expression [18]. In Lake Victoria cichlids of the genus *Pundamilia*, different species and populations show different ratios of LWS/SWS expression which correlate with variation in light environment [14]. These expression patterns were examined using laboratory fishes bred and kept under identical light conditions. It is hence likely that the differences in opsin expression patterns are heritable. In our model framework, a wide range of possible gene control modes were permissive of speciation. Even if expression patterns could not evolve, speciation was possible. On the other hand, speciation was impossible if the wavelength of peak absorption could not evolve. This may suggest that spectral tuning by changes in the peak absorption of opsin genes is more important for sensory drive speciation than that by changes in the relative extent of the expression of different opsin genes. This might be because mutations causing a change in the peak absorbance might be more likely to cause divergent changes in the peak spectral sensitivity. However, this result may change if trade-offs between the expression of different opsin genes are assumed.

Second, at present, selection intensity for adaptation to environmental light regime is unknown. In our model, increasing the strength of selection intensity (decreasing V value) led to an increased probability of speciation. In the simulations with the core set of parameter values, V was set to 100. In our model, V = 100 means that individuals suffer a 25% fitness reduction if they are 25% less effective at perceiving the predominant environmental light than individuals with the optimum perception. We also simulated with V = 400 and otherwise the core set of parameter values. The probability of speciation was the same as in the simulations with V = 100. For V = 400, individuals suffer only a 6% fitness reduction if they are 25% less effective than individuals with the optimum perception. Thus, the assumed strength of selection might not be unrealistically large [70].

Finally, we considered background light as identical with environmental color. This is a simplification that we think is justified if the background that females see courting males against is the water column, or has a color similar to that of the water column.

### Conditions for speciation

We observed that in a large part of the parameter space the probability of speciation increased with decreasing dispersal distance and size of the mating area, and with increasing local carrying capacity, population size and relative strength of female preference (α). Small genetic neighborhood size (small individual dispersal distance and mating area) promotes the buildup of associations between mating loci (here male color alleles) and fitness related loci (here opsin gene alleles; [71]). Increased densities increase the speciation rates probably because increasing the number of candidate males within the mating area of a female increases the effectiveness of female choice [72]. In our model, with K = 10, about 1000 individuals (500 males) were maintained in the 1000 × 1000 cells habitat. If we assume that the length of the slope between the surface and the deepest end of the habitable area (y-axis = 1000) is about 40–50 m and male territory size is about 3 m^2 ^(in *Pundamilia*, male territory size is about 3 m^2^, [11]), the number of territorial males would be about 530 to 830 within the 1000 × 1000 cells habitat. Thus, local carrying capacity used in the model was not unrealistic.

Increasing population size by increasing habitat size along the horizontal axis caused an increased probability of speciation. This might be because selection becomes more effective in larger populations. Importantly, the increase in the speciation rate with increasing habitat size is not due to an increased opportunity for isolation by distance because the axis of increased opportunity for isolation by distance was perpendicular to the selection gradient along which incipient species diverged. In this aspect our model differs fundamentally from classical models of clinal speciation, in which selection and isolation by distance reinforce each other [7,52,58,73].

For mutation rates (μ), we usually assumed 10^-5 ^for loci for male nuptial coloration and loci controlling the amount of opsin gene expression. These might be reasonable values. On the other hand, the rates of mutations causing change in absorption spectra of opsin genes might be 10^-8 ^or lower. In our simulations, the probability of speciation was 40% for μ = 10^-6^, and 5% for μ = 10^-7 ^when population size was about 900. However, increasing the population size increased the probability of speciation, and speciation occurred even with mutation rates as low as 10^-8 ^when the population size was sufficiently large (Fig. 2d). In addition, in most of our models, we simulated 5,000 generations, but when simulations ran for 10,000 generations, the probability of speciation increased 1.2-fold (results not shown). Thus, lower mutation rates caused both decreasing the probability of speciation and delaying speciation.

Speciation was slightly more likely with the ''100-additive-loci model'' and with a ''one major epistatic locus model'' for male nuptial color, than with the ''5-additive-loci model''. Empirical observations on the genetic control of color patterns in cichlid fishes suggest that major dominant gene effects may not be uncommon [74,75]. However, compared to that of female mating preference, genetic control of male ornamentation had only a minor effect on the probability of speciation.

The width of the reflectance spectra of male nuptial coloration (σ_m_) did not affect the results when σ_m _was 10 or smaller (i.e, about 60 nm or less). Above 60 nm spectral width, the probability of speciation decreased with increasing spectral width. However, speciation frequently occurred even when σ_m _was 16 (i.e, about 100 nm) (Fig. 3c). A spectral width of 40 nm (σ_m _= 6) corresponds to the highest 10% of actual reflection spectra of male nuptial colors in Lake Victoria cichlid fish [15]. A spectral width of 100 nm corresponds to the sections of the reflectance spectra the intensities of which do not overlap between red and blue species [15].

We find that the relationship between the probability of speciation and the width of the predominant light spectrum (σ_E_) is similar to its relationship with the width of the reflectance spectrum of male nuptial coloration (σ_m_). Speciation is frequently observed even with a spectral width of 100 nm (σ_E _= 16, Fig. 3c). The width of aquatic light spectra depends on the clarity of the water. In the clear waters of Lake Victoria, the widths of ambient light spectra at 1 and 9 m under the surface are approximately 1000 and 110 nm, respectively. A width of the predominant light spectrum of 100 nm corresponds to the highest 10% of the ambient light spectra at 1 m under the surface and to the highest 90% of ambient light spectra at 9 m under the surface [15]. In less clear water, 100 nm can correspond to 90% or more of the width of the ambient spectrum even at only 2 m under the surface [54]. It may be interesting to note that in sticklebacks, different visual sensitivities have evolved even where the difference in the peak wavelength of the water color was only 20 nm [8].

Recently several authors have concluded, based on simulation studies, that sympatric speciation can more easily occur than previously thought [40,41]. Gavrilets [47] indicated that these simulations may have been unrealistic because the authors assumed high genetic variation for all traits, and/or high mutation rates. In our simulations, female preference was initially invariable, and only one or two of up to 100 loci for male nuptial coloration were polymorphic. In addition, we also did simulations starting from no initial genetic variation for male nuptial coloration at all (i.e., the results for our one locus major gene model), which yielded similar speciation probabilities as our other models. Hence our simulation results are robust to, and independent of, variation in standing genetic variation.

### Implications and predictions of the models

**Figure 4:**
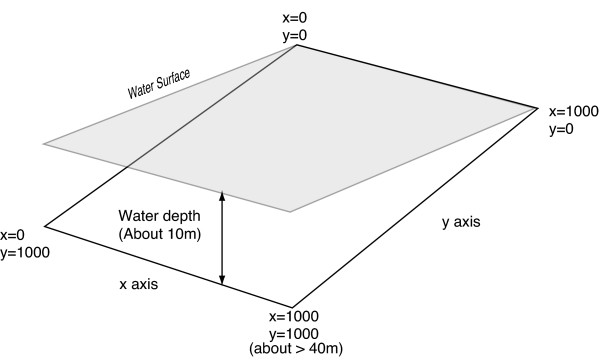
**The simulation area and supposed habitat size. The simulation was conducted in 1000 × 1000 square area**. This was assumed as a slope of the bottom of a lake.

Our results provide several new and testable predictions for when sensory drive is likely or unlikely to cause speciation within a continuous population. In our simulations speciation occurred most frequently when environmental light gradients were of intermediate steepness and dispersal distance of offspring was relatively small. Because we kept the length of the environmental light gradient (G_E_) constant, the variation in steepness of G_E _was synonymous with variation in the magnitude of the selection differential between the ends of the cline (e.g. the depth difference between the ends of the y axis, see Fig. 4). Lack of speciation at G_E _< 0.1 was likely due to a too small selection differential. Lack of speciation at larger dispersal distances is due to the breakdown of local adaptation (Hendry et al. 2001). The steeper the selection gradient the smaller the dispersal distance that is sufficient to break down local adaptation. This may explain the counterintuitive result that speciation is not common when the selection gradient is very steep. In addition, small dispersal distances cause spatial clumping of related genotypes, which may promote the establishment of linkage disequilibrium between mating preferences and male secondary sexual traits and hence speciation [71,76].

Values of G_E_, that were permissive of speciation, are not unrealistic: When the environmental light gradient (G_E_) was 0.1, the difference in the mean environmental light color between upper and lower end of the habitat was 100 nm. In Lake Victoria, the mean environmental light color can range from 500 nm near the surface to 590 at between 2 and 10 m water depth, depending on the turbidity [10]. Interestingly, sympatric species are better differentiated where this gradient is less steep, and differentiation breaks down where the gradient is extremely steep, e.g. in highly turbid waters [10], consistent with the results of our simulations.

Possibly one of the most interesting results of our simulations is that speciation never occurred when we assumed a stepwise mutation model for the opsin genes (steps of 1 nm), whereas speciation was frequent when we used a 5 sites mutation model designed after Yokoyama and Radlwimmer's empirical "five-site rule" for the molecular basis of visual tuning [19]. This indicates that mutations causing major changes in female preference promote speciation. If individual mutations cause only minor changes in phenotypes, gene flow and recombination might prevent adaptation to local environments [77]. In our model, female mating preferences were determined by differences in the peak absorbance of the 3 opsin proteins. In many previous speciation models, the relationships between genotypes and phenotypes assumed additive effects. The genetic system modeled by us might not apply to organisms in which the sensory drive is associated with non-visual sensory modalities. However, polygene control with small additive effects might not be the general genetic architecture of ecologically important phenotypes (e.g., [78]), and the role of major gene effects in speciation may be of more widespread importance. This should be tested in future studies. Thus, our results on explicitly modeled speciation through vision-mediated sensory drive indicate that detailed information about the genetic control of traits is required to predict the probability of speciation via a given hypothetical mechanism.

### Comparison with previous models

That environmental clines may result in parapatric speciation is a classical scenario [73]. Our models are similar to the model of Doebeli and Dieckmann [58], in which a spatial gradient was assumed, along which the relative fitness of competing genotypes varies. Our model differs in that the environmental gradient is a lot shorter relative to dispersal distances, and is perpendicular to the axis of maximum opportunity for isolation by distance, making our model applicable to empirically characterized cases of speciation within single habitat patches, referred to as sympatric. In their models, a spatial selection gradient coupled with non-random mating with regard to ecological traits, created conditions favorable for speciation without geographical barriers. However, like in the sympatric models, the authors had to assume much higher mutation rates than we did (10^-3 ^per locus per generation) and high initial genetic variation [47], both of which might be unrealistic.

In our genetically explicit sensory drive speciation model, individual sensory properties are associated with both environmental adaptation and female mating preference. Such association between two traits facilitates speciation [36,47]. In some earlier models, the cost of mate choice prevented speciation in such scenarios [47,71]. In our model, a female chooses the male among all males within her mating area whose nuptial color signal she is most sensitive to. Females easily find and compare mates since they are conspicuous and occur at high density. In this situation, there might be little or no cost of female choice. We assumed female choice incurs a searching cost that is proportional to the distance that a female has to travel to make her choice. Our results suggest that the probability for speciation, given otherwise our core parameter set, was 0.9 and 0.6 even when these costs depressed female preference for males near the edges of a female's mating area by 20 % and 30%, respectively. This compares to a probability of 1 when costs were zero. Thus, search costs did not significantly affect the results of this study.

Several studies [43-45] proposed that 2 simultaneous runaway processes can cause sympatric speciation by sexual selection. In the same population, if some females prefer red males and others prefer blue males, then runaway sexual selection may be triggered, and it may create a pair of species – one with red-preferring females and red males and another with blue-preferring females and blue males. Recent studies [48,49] indicated that sympatric speciation only through female choice sexual selection is unlikely, unless the initial genetic variance in female preference is large and also symmetric. The probability of speciation in our model was neither significantly affected by the initial mean opsin protein absorption spectrum and associated female mating preference nor by their variance, unless the initial sensitivity curve (e.g., Fig 3a) was prominently bimodal or multimodal, which enhanced the probability of speciation. Hence, speciation in our model did not require symmetric distributions of genetic variances as the starting condition. On the other hand, when the light environment was spatially homogeneous or when adaptation to light environments was not allowed, speciation did not occur. In addition, when no selection for spectral sensitivity (i.e., (E_max _- P)^2^/V = 0 in the fitness formulation) was assumed in our model, speciation did not occur. In the simulation runs without assumed selection for spectral sensitivity, some variations in male colors (a range of peak color = 30 nm) and female preference (a range of peak sensitivity = approximately 30 nm) were maintained across 5000 generations; however, speciation was not observed, even if the mutation rates were set high (μ = 0.001). These results indicate that visual runaway sexual selection alone is unlikely to drive sympatric speciation in situations without or with low genetic variations.

## Conclusion

We showed for the first time that in simulations in which the adaptive evolution of visual pigments and color perception are explicitly modeled, sensory drive can promote speciation along a short selection gradient within a continuous habitat and population. We conclude, in a homogenous environment, speciation by female choice sexual selection alone is unlikely unless other mechanisms are in place [46]. On the other hand, it may not be unlikely where environmental gradients create divergent selection on sensory modalities that are used in mate choice. Evidence for such exists from several animal groups [1,79,80]. The probability of speciation in a continuous population under such conditions may then critically depend on the relationship between size and steepness of the selection gradient and dispersal distance on the one hand, and on the genetic architecture of adaptation in the sensory system and female mate choice on the other hand.

## Methods

### Life history

We simulated a population inhabiting a single habitat patch. Each individual has a location (*x*-*y *coordinates), sex and reproduces sexually (diploid) ; and each has a set of alleles at loci for male nuptial color, opsin genes and loci controlling opsin gene expression. Individuals forage and reproduce in local environments that vary in ambient light color along a continuous gradient. Their survival probability is affected by the goodness of the match between their visual properties and the predominant environmental light color, e.g. as if the latter affected the ease with which food items are detected. Females choose mates among the males within a mating area of a given diameter. The probability that a given male mates with a given female depends on her visual sensitivity to his nuptial coloration and on the number of competing males with similarly well or better matching nuptial coloration in the female's mating area. The number of offspring produced by a female is determined by the number of competitors for resources within her home range (local density) and by the match between her visual properties and the environmental light color. Offspring disperse from their mother's site (see below). All parents die after the offspring is produced so that generations do not overlap. Individuals hold territories and are initially randomly distributed within the habitat.

### Habitat and light environments

**Figure 5:**
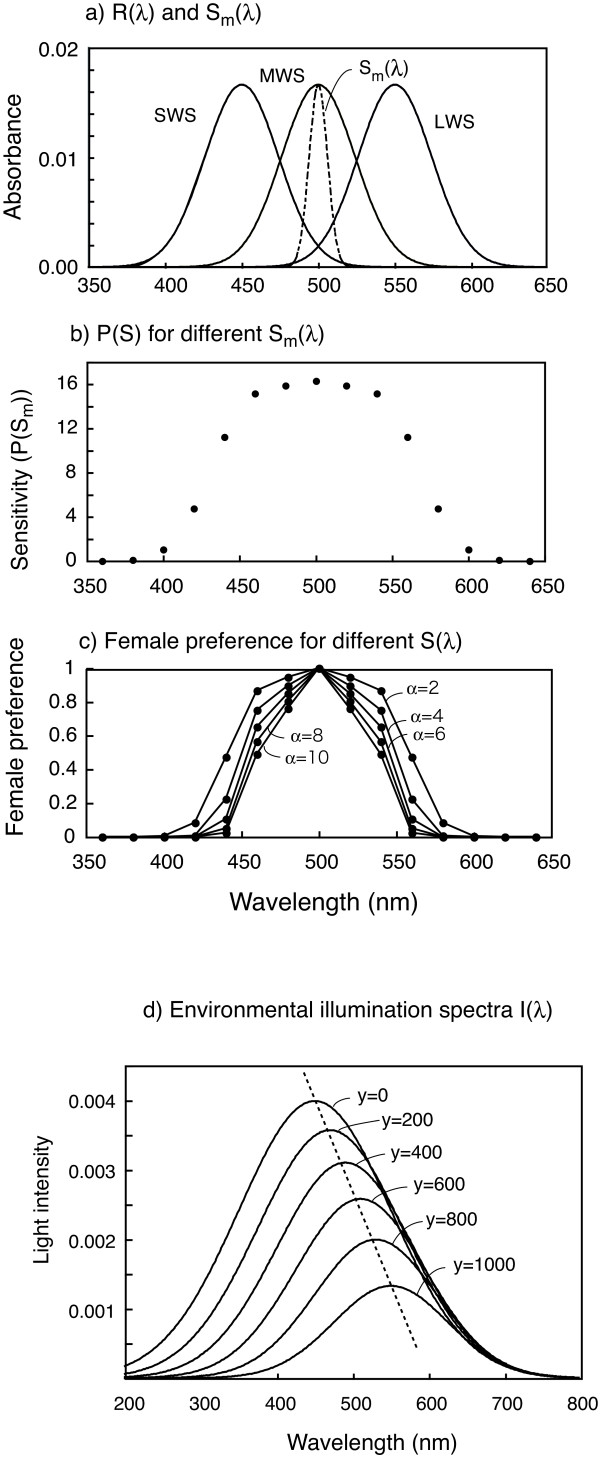
**Sensitivity spectra of three photoreceptor cells with different visual pigments (three solid curves = R(λ)) and reflectance spectrum of a male nuptial color (a broken curve = S_m_(λ)) (a), visual sensitivity (b), female preference for male coloration (c), all in the initial condition, and environmental illumination spectra at different depth (d)**. a) The peak wavelengths of R(λ) for SWS pigment alleles (λ_B1 _and λ_B2_) are 450 nm, those of MWS (λ_G1 _and λ_G2_) are 500 nm and those of LWS (λ_B1 _and λ_B2_) are 550 nm. The peak wavelength of S_m_(λ) is 500 nm. b) The total visual sensitivity for 15 different S_m_(λ) is calculated as zP(S_m_), where zP(S_m_) is calculates by using equation 2 and z is constance (see text). c) Relative strength of female preference to 15 different S_m_(λ). Values for female preference are relative values when the maximum F was set to 1. d) A broken line indicates a gradient of predominant wavelength of light along depth. y is vertical axis of the simulation space, and spatial point at y = 1000 corresponds to the water depth of about 10 m (Fig. 4).

The evolutionary dynamics take place within a grid of 1000 × 1000 cells (Fig. 4), with a gradient in the ambient illumination spectrum (I(λ)) along the y axis. We can think of this as the floor of a lake or sea with a gradient of ambient light from shallow near-shore to deep. The ambient light color in water varies with water depth. Both, the steepness and direction of the resulting gradients are determined by the amounts of organic and inorganic matter dissolved and dispersed in the water. Whereas in clear water, such as in oligotrophic lakes and the oceans, longer wavelengths are attenuated with increasing depth more quickly than shorter wavelengths (and the ambient color at greater depths is blue), in eutrophic and dystrophic lakes blue wavelengths are attenuated most quickly with depth, and thus the ambient color in deeper water is red-shifted relative to shallow water [60,81]. In our model the predominant wavelength in the ambient light shifts upwards and the light intensity decreases with increasing depth (Fig. 5d).

We parameterized the light gradient such that it resembled that from 0 to 10 m depth in a mesotrophic freshwater lake, e.g., offshore Lake Victoria [10,15]. Oligotrophic gradients would be shallower, eutrophic gradients steeper. The ambient illumination spectra (I(λ)) were assumed to conform to a normal distribution with mean (E_c_) and standard deviation (σ_E_). E_c _represents the predominant wavelength in the ambient light, which changes along the y axis in our model space. Then, E_c _(in nm) and σ_E _are given by

E_c _= 500+G_E_(y-500)

and

σ_E _= 87.5-G_s_(y-500)

where y is the vertical position of the individual and G_E _and G_s _are the steepness of the gradient in predominant light color (peak wavelength), and of the decrease in the width of ambient spectra, respectively. Light intensity is given by

E_I _= 6.25-G_I_(y-500)

where G_I _is the steepness with which light intensity decreases with depth. When the light intensity is considered, the ambient spectrum thus becomes E_I_I(λ)(thereafter, I(λ) = E_I_I(λ)). We varied the value of G_E _(0 – 0.225) to examine the effect of environmental gradient steepness on the probability of speciation. G_I _and G_s _were usually set to 0.0075 and 0.025, respectively, and this parameter setting and the gradient formula (G_E_, σ_E _and E_I_) creates the environmental illumination shown in Fig. 5d which resemble the environmental light spectrum observed in Lake Victoria [15]. Changing these values (G_I _and G_s_) did not significantly affect the results.

### Spectral sensitivity and visual perception

Spectral sensitivity (luminous efficiency function) is given by the minimal intensity of monochromatic light of given wavelength that can be detected. We assumed that how easily an individual detects light of a given wavelength depends on the spectral sensitivity which is determined by the absorption spectra of its visual pigments (opsins), and its patterns of differential opsin gene expression [14,62].

Perception of luminance of any item is related to the quantum catch of the cone cells. For a given photoreceptor i, the quantum catch of the cell is given by

Q_i _= ∫ I (λ)S(λ)R_i_(λ)dλ

where I(λ) is the illumination spectrum (i.e. the spectrum of the down-welling light), S(λ) is the reflectance spectrum of the male nuptial color, and R_i _(λ) is the absorption properties of the photoreceptor i [82]. The transmission of reflected light from the male to the female was not modeled since males move around the female during courtship, and the distance between the male and the female was assumed to be constant. I(λ) varies with water depth as shown in the previous section.

Receptors physiologically adjust their sensitivity to the intensity of light they absorb. After this light adaptation, the quantum catch q_i _of each receptor adapted to its background is,

q_i _= a_i_Q_i_,

where a_i _is the von Kries adaptation coefficient and can be described as

a_i _= 1/∫ I(λ)R_i_(λ)dλ

[83,84].

The brightness or luminance signal was additively collected from the photoreceptor cells [85,86]. Perceived luminance of an object is the sum of the quantum catches of all photoreceptor cells. The relative contribution to the sensitivity differs depending on cone types. We assumed that an individual has three opsin genes, SWS (short-wave sensitive), MWS (middle-wave sensitive) and LWS (long-wave sensitive), each of which is expressed in a different class of cone cells. The relative contributions of the three classes of cone cells to overall sensitivity depend on the relative amounts of expression of the three opsin genes, which could be related to the number of cells per cone class, and on the amount of gene expression within a cell, as well as on relative strength of electric signal from cells of each cone class. Then, total sensitivity for an object with reflectance spectrum (S) after light adaptation to background lights is

P(S) = k_R _(q_R1 _+ q_R2_) + k_G _(q_G1 _+ q_G1_)+ k_B _(q_B1 _+ q_B2_)

where q_R1_, q_R2_, q_G1_, q_G2_, q_B1 _and q_B2 _represent quantum catches of cells with opsin pigments coded by allele 1 and 2, of the LWS, MWS and SWS locus respectively using equation (1). k_R_, k_G _and k_B _represent the relative contributions to overall sensitivity by LWS, MWS and SWS cone opsins [87]. k includes the relative strength of electric signal from each cone type and the relative amounts of LWS (R), MWS(G) and SWS(B) opsin gene expression. For calculation of q_R1_, q_R2_, q_G1_, q_G2_, q_B1 _and q_B2_, R_R1 _(λ), R_R2 _(λ), R_G1 _(λ), R_G2 _(λ), R_B1 _(λ) and R_B2 _(λ) were used as the absorption spectra of the different visual pigments. λ_R1_, λ_R2_, λ_G1_, λ_G2_,λ_B1 _and λ_B2 _are the peak absorption wavelengths of allele 1 and 2, of the LWS, MWS and SWS locus respectively. The absorption spectra of the corresponding cones R(λ), each coded by a single opsin gene with two alleles, were assumed to exhibit a normal distribution (Fig. 5a) for simplicity (standard deviation of the distribution was set at 24), although the exact shape of absorption spectra of visual pigments has a shoulder. The initial peaks of the absorption spectra (λmax) of SWS, MWS and LWS opsin were set at 450, 500 and 550 nm respectively and initially no genetic variation in peak wavelength was assumed. For Lake Victoria cichlids of the genus *Pundamilia*, the λmax of three photoreceptor classes are 455, 528 and 565, and there seems to be little variation in λmax within a population [14].

### Genetic control of variation in visual perception

Heritable variation in spectral sensitivity was assumed to be caused by allelic variation at three opsin genes and by polygenic additive variation for k (relative strengths of electric cone signals and/or relative amounts of LWS, MWS and SWS opsin gene expression). Mutations at each opsin locus causes a change in the absorption spectrum.

At present, there is no information as to how k (relative strength of electric signal and/or relative amounts of LWS, MWS and SWS opsin gene expression) is genetically controlled. Thus, we explored a series of different models for determining k. (1) *10-locus model*: k of each opsin gene was assumed to be controlled by 10 additive regulatory loci. Each locus had three alleles (-1, 0 and 1). Initially, allele 0 was fixed at all loci, and then k = 1. Allele 1 and allele -1, respectively increased or decreased perception intensity by 0.04 and thus, when allele 1 was fixed at all loci that control k of one opsin gene, perception intensity (k) became 1.8. This model was used unless specified otherwise. (2) *5-locus model*: k was assumed to be controlled as above except that only 5 additive loci, each with the effect of 0.08, controlled each opsin gene. (3) 3- *locus model*: as above except that only 3 additive loci each with the effect of 0.14 control k. (4) *No differential expression and no electric signal change model*: k was set to 1 and was kept invariable. (5) *One locus controlling on-off switch model*: One locus controlled k of each opsin gene. A mutation to that locus caused loss of function of the opsin gene (i.e., k = 0; e.g. through loss of expression). Reverse mutation was allowed. (6) *No peak shift model*: We used our 10 loci model for k, but did not allow any mutation to the amino acid sequences of the opsin genes. Hence, the absorption spectra of individual opsin genes were kept invariant.

Additional to these unconstrained models, we also conducted simulations in which the minimum distance between the absorption peaks of LWS, MWS and SWS was constrained. These simulations modeled the case where selection (e.g. for detection of prey by contrast against the predominant background light) would act to retain trichromatism. Trichromatism is common in fishes of freshwaters and shallow seas [60]. We simulated minimum distances of 10 nm and 20 nm. Finally, we studied in addition to the "3 opsin genes models", a "4 opsin genes model". The initial peak absorption wavelengths of 4 opsin genes were 400, 450, 500, and 550 nm. We studied effects of variation in gene expression in this model with the 10 loci per opsin gene, described above for the *3 opsin genes model*, as well as with the *one locus on-off switch model *(expression level 0 when the genotype of the locus was 0–0).

### Mutation models

Analysis of 11 vertebrate MWS and LWS opsin pigments indicated that five different single amino acid substitutions S180A, H197Y, Y277F, T285A, A308S, and the double mutation S180A/H197Y shift λmax by -7, -28,-8, -15, -27, and 11 nm, respectively [19]. Yokoyama and Radlwimmer [19] suggested that this "five-sites rule" is the molecular basis of spectral tuning in the MWS and LWS pigments. We modeled this five sites rule and assumed that 5 mutations shift the λmax of the pigments by ± 7, ± 28, ± 8, ± 15, and ± 27 nm. The double mutation was not considered in the model, but its effect might be similar to those of single mutations with higher mutation rates. In addition, it is reasonable to assume that unknown single site mutations shift the λmax of the pigments slightly. Thus, we assumed that additional single site mutations shift the λmax by ± 1 nm. We call this mutation model *5 sites mutation*. To examine the effect of mutation mode on the results, we also did a simulation in which all mutations shifted the λmax of the visual pigments only by ± 1. We call this mutation mode *stepwise mutation*.

Mutation rates (μ) of genes for male nuptial coloration and for the amount of opsin gene expression were assumed to be 10^-5 ^per locus per generation. For 5 amino acid sites in which a mutation causes a shift of the λmax (± 7, ± 28, ± 8, ± 15, and ± 27 nm), a mutation occurs independently for each site and the mutation rate of 10^-5 ^per generation per site was assumed in simulations unless specified otherwise. For unknown sites causing a shift of ± 1 nm, the mutation rate of 10^-5 ^per generation per all these sites was assumed. For each site, when a mutation occurs, the probability of negative or positive shift of the λmax was 0.5. To examine the effects of mutation rates in the amino acid sequence of opsin pigments, simulations with μ = 10^-3^,10^-4 ^10^-5^,10^-6^,10^-7 ^and 10^-8 ^were also conducted. In the stepwise mutation model, only mutations causing a shift of ± 1 nm occur at each opsin locus. Simulations with μ = 10^-3^,10^-4 ^10^-5 ^and 10^-6 ^were conducted.

### Environment-dependent female fitness

We assumed that individuals that can see the predominant light color of the ambient light better have higher fitness. The predominant ambient light spectrum (S_e_) was assumed to conform to a normal distribution where the mean and variance are E_c _(i.e., the peak of ambient illumination spectrum) and σ_e_, respectively. σ_e _is smaller than σ_E_, and thus, S_e _is a predominant part of the ambient illumination spectrum (I(λ)). How sensitive individuals are to the predominant ambient light depends on the perception sensitivity to the predominant light spectrum (S_e_(λ)) at a given environmental illumination spectrum (I(λ)).

Perception sensitivity (P(S_e_)) to a predominant ambient light was calculated using equations and (1) and (2) (see the section of *Visual perception for lights*).

P(S) = k_R _(q_R1 _+ q_R2_) + k_G _(q_G1 _+ q_G1_) + k_B _(q_B1 _+ q_B2_)

where q_R1_, q_R2_, q_G1_, q_G2_, q_B1_, and q_B2 _represent the quantum catches of cells with opsin pigments coded by alleles 1 and 2 of the LWS, MWS, and SWS locus, respectively (see *Spectral sensitivity and visual perception*).

q was calculated as

q_i _= a_i_Q_i_,

where a_i _is the von Kries adaptation coefficient and Q_i _= ∫ I(λ)S_e_(λ)R_i_(λ)dλ.

I(λ) is the illumination spectrum (i.e., the spectrum of the downwelling light), S_e _is the predominant ambient light spectrum, and R_i _is the absorption spectrum of cells with opsin allele i. When σ_e _(the width of the predominant light spectrum) is set to 10, the width of the reflectance spectra becomes approximately 60 nm. During simulation, σ_e _was usually set at 10, but the effects of varying σ_e _(4–16, corresponding to 20–100 nm) were examined. When σ_e _is set to 10, the width of the reflectance spectra become about 60 nm. In the simulation, σ_e _was usually set at 10, but the effects of varying σ_e _(4–16, corresponding to 20–100 nm) were examined.

The fitness of an individual was described as follows:

W = 2 + (1 - N/K)r - (E_max _- P_f_)^2^/V

where r = reproductive rate (r = 1.6); N = number of individuals within the home range (radius of the home range = 50); K = carrying capacity within a female's home range; E_max _= maximum perception above which perception intensity cannot increase (E_max _= 22); P_f _= zP(S_e_), where P(S_e_) is the sensitivity for the predominant light spectrum (S_e_) and z is a constant; and V = stabilizing selection intensity (V = 100). Because P(S_e_) is usually less than 1, z adjusts P(S_m_) values to greater than 1 for calculation purposes (z = 1500, changing this value does not affect the results). The minimum value of W was set at 0. N was counted as the number of adult individuals within a circle of radius 50 around a focal individual. The calculated W value was converted to an integral value; for instance, when W was 1.6, it was converted to 2 with a probability of 0.6 For a female, W was the number of offspring she gives birth to. Males died before the reproduction when W = 0. The intensity of selection for local adaptation in the visual system increases with decreasing V. The first term in the equation giving fitness, represents "baseline fertility", and the second term describes density-dependent selection. The last term describes selection for visual adaptation to the predominant light color at the site where the individual was located, and this formula is the same as the equation describing adaptation to local environments along a spatial gradient [88]. P_f _values vary with environmental light conditions and with the spectral sensitivity of an individual and thus, the individual increases its fitness when its spectral sensitivity adapts to effectively perceive the predominant light color at the site where it lives. The P_f _value at the wavelength of the peak spectral sensitivity was initially approximately 15, and the maximum value of P_f _increased through evolution and approached the E_max _value. The E_max _value was set at 22 since we assumed that the maximum sensitivity increases by 1.5-fold from the initial sensitivity value. This value was rather arbitrary, but varying E_max _values ranging from 20 to 28 did not affect the results. With R set at 1.6, approximately 1000 individuals were maintained in a 1000 × 1000 habitat when K = 10. This is likely to be a strong underestimation of the typical population size of the cichlid fish of Lake Victoria, but it is a realistic value of density (see Discussion). An increase in r increased local densities that affected the speciation rates. Thus, we examined the effects of local densities on speciation rates by varying carrying capacity (K ranged from 5 to 14). At the start of simulations the population was assumed to be adapted to intermediate water depth (however, we also modeled situations where the initial population was at either extreme of the spectrum.

### Male nuptial coloration

Male nuptial color was controlled by 100 genetic loci, each with two alleles (1 and 0)(*100-additive-locus model*). When allele-0 was fixed for all loci, the reflection spectrum of the male body color peaked on 400 nm (blue), whereas when allele-1 was fixed for all loci it peaked on 600 nm (red). Initially, two loci were polymorphic so that the peak wavelength of male nuptial color ranged from 498 to 502 nm. The reflectance spectrum of a male was assumed to be a normal distribution (Sm(λ)) with standard deviation (σ_m_) and mean (the peak wavelength of male nuptial color). When σ_m _is set at 6, the width of the reflectance spectrum of male coloration becomes about 40 nm. In the simulation, σ_m _was usually set at 6, but the effects of varying σ_m _(4–16, corresponding to 20–100 nm) were examined.

Additionally, in a second set of models, we assumed that male nuptial color was controlled by only 5 loci which is close to observations on some cichlid fishes [30,74]. Two different 5-loci models were investigated. In the first model, the effects of alleles at all 5 loci were additive (5-*additive-locus model*). Each locus had two alleles (1 and 0), and allele-1 increased the wavelength of the color by either 5, 10, 15 or 20 nm (four separate models). For instance, when allele-1 increased the wavelength by 5 nm, the wavelength of nuptial body color ranged from 475 to 525 nm. Initially, one locus was polymorphic so that the initial wavelength of male nuptial color ranged from 495 to 505 nm.

In the second 5-locus model, the effects of alleles at four loci were additive, but a fifth locus had a major effect on the color and allele 1 was dominant at this locus (*one major epistatic locus model*). Each of the four additive loci had two alleles (1 and 0), and allele 1 increased the wavelength of the color again by either 5, 10, 15 or 20 nm. When allele 1 was present at the major locus, the nuptial body color was determined only by this locus and reflected at long wave lengths irrespective of the alleles at the additive loci. When allele 0 was fixed at the major locus, allele 1 at any of the additive loci increased the wavelength by 5 nm, the wavelength of the nuptial color ranging from 480 to 520 nm. When allele 1 was present at the major locus (hetero- or homozygous), the wavelength of nuptial color was 530 nm irrespective of the alleles at the four additive loci. Initial body color of all males was 500 nm, none of the additive loci were polymorphic, and allele 0 was fixed at the major locus. No linkage was assumed among any of the loci.

In addition, we conducted simulations with the one major epistatic locus model in which both spectral tuning and male nuptial color were initially either shifted to long-wave color (λ_B _= 510, λ_G _= 560, λ_R _= 610 nm, male nuptial color = 560 nm) or to short- wave color (λ_B _= 390, λ_G _= 440, λ_R _= 490 nm, male nuptial color = 440 nm).

### Mating and female mating preference

The sensory drive hypothesis predicts that females evolve to actively prefer those male courtship signals that they can most easily detect because close matching of female detection ability and male signal increases the effectiveness of communication [1,2,89]. Thus, we assumed that females actively prefer males the nuptial coloration of which reflects at wavelength that they detect most easily, and that females detect male signals easily if the sum of the male's reflectance × the female's sensitivity is large. Hence, females evolve to actively prefer males that reflect most strongly on the mode of the female sensitivity function. Thus, the strength of female preference for males with reflectance spectrum (S_m_) was

F(S) = (zP(S_m_))^α^,

where P is the sensitivity function described above, α is the strength of female preference relative to perception intensity and z is a constant. Because P(S_m_) is usually less than one, z adjusts P(S_m_) values to larger than one for calculation purposes (z = 1500, changing this value does not affect the results, see also *Environment-dependent female fitness*).

A female chooses her mate among all males within a female's mating area with a radius (M). The probability that a male with nuptial reflectance spectrum (S_m_) mates with a given female then is

F(S_i_)/ΣF(S_j_)

where F(S_i_) = the strength of female preference for male *i*, determined by the reflectance spectrum of the male's body color (S_m_), and Σ F(S_j_) = the sum of the strength of the female's preference for all the males within her mating area. Figs. 5a and 5b illustrate the spectra of female visual pigments (R) and a male's body color (S_m_) and the total female sensitivity function, respectively. Fig 5c illustrates the corresponding female preferences to different peaks of S_m _(preference function) at different α. The female preference function is the female's relative liking for male colour of a given wavelength when the strongest liking is set to 1. With increasing α, females are more likely to mate with males with the nuptial color that is most intensely perceived.

### The cost of female choice

The cost of mate choice can prevent speciation [47,71]. The cost of female choice includes the expenses for search and discrimination time, increased exposure to predators, energy expenditure and lost opportunity. In our model, females choose the male the nuptial signal of which they are most sensitive to among all males within a female's mating area. Females easily find and compare mates since they are conspicuous and occur at high density. In this situation, there might be little or no cost associated with female choice. However, we consider that females incur search costs that are proportional to the distance of a male from the center of a female's mating area so that females are less likely to choose more distant males. The cost is

f = 1-cD^2^

where D is the distance of the male from the center of the female's mating area, and c is a coefficient of the cost. Then, the strength of female preference for the male is reduced by f and thus, fF(S_i_) was used instead of F(S_i_) for calculating the probability of matings. We varied c values from 0 to 0.00005. When c = 0.00003 and D = 100 (i.e., this is equivalent to the value for the radius of female mating area 100 cells in a 1000 × 1000 cell grid), the relative strength of female preference for the male is reduced by 30%.

### Dispersal and neighborhood size

Offspring dispersed from their birth sites. The dispersal distance (D) was a random value drawn from a normal distribution with mean 0 and standard deviation d (negative values were converted to positive values). Dispersal direction was a random number ranging from 0 to 2π. The dispersed site of the offspring (x_o_, y_o_) was calculated as x_o_=round(x_m_+con(angle)*D) and y_o_=round(y_m_+sin(angle)*D), where x_m_, y_m _is the location of the mother, and round(v) means the integer number that is closest in the value v. Individuals could not disperse to outside of the habitat patch. Thus, a dispersal site of an offspring was recalculated until the site was located within the habitat.

### Simulation sequence

The program was written in C++ and the codes were compiled by Xcode on Macintosh. The simulation program was conducted in the following order. (1) 500 individuals were created as the initial population (for 1000 × 1000 habitat); (2) The number of adult individuals was counted within the home range of individual i; (3) The sensitivity to the spectrum of predominant light in the environment was calculated for individual i; (4) The fitness was calculated for individual i; (5) The sensitivity to nuptial color reflectance spectra of males located within the mating area was calculated for individual i when the individual was female; (6) The female choose one male within the mating area. The male with nuptial color whose reflectance spectrum best matched the female's visual sensitivity had the highest probability to be chosen by the female; (7) The female produced its offspring, the number of which depended on her fitness; (8) The offspring dispersed from their mother's site; (9) The procedures (2)–(8) were repeated for each individual. (10) All adults died; (11) the procedures (2)–(10) were repeated for 5000 times.

### Parameters examined

Table 1 summarizes the parameters used. In most of our simulations, the average peak reflectance of male nuptial color was initially set to 500 nm as described above, and females perceived the color of 500 nm most intensely (Fig. 5a). However, we also simulated situations in which reflectance and perception were initially at either end of the spectrum. All models started with monomorphic opsin gene sequences, and so maximum perception of all females was at the same wavelength.

Five hundred individuals existed at the beginning of each simulation when habitat size was 1000 × 1000. After 5000 generations, we examined whether male nuptial colors and female preference had diverged into two distinct phenotype groups and also whether such subpopulations with different nuptial colors were reproductively isolated from each other. Ten or twenty replicate simulations were conducted for each parameter combination, and the different parameter combinations were obtained by varying (i) the steepness of the environmental gradient in predominant light colour (G_E _= 0–0.225), (ii) the relative strength of female preference(α 1–14), (iii) dispersal distance (*d=*50–225), (iv) size of mating area (M, 75–300), (v) mutation mode (5 sites mutation model and stepwise mutation models), (vi) mutation rate(μ, 10^-3 ^– 10^-8^), (vii) habitat size (x axis; perpendicular to the direction of the selection gradient, y = 1000, x = 1000–5000), (viii) carrying capacity (K, 5–15), (ix) different models for the inheritance of male coloration (one major-epistatic-locus model and 5-additive-locus model), (x) 8 different models for the inheritance of opsin gene expression, (xi) the width of the reflectance spectrum of male coloration and the width of the predominant ambient light spectrum (σ_e _= 6–16, and σ_m _= 6–16), (xii) the cost of mating preferences (c = 0–5). (i) and (ii) affect the strength of natural and sexual selection. (iii) and (iv) affect the gene flow across the habitat. (vii) and (viii) affect population size and density.

Simulations with parameter values used for the environmental light (I(λ), E_c_, E_I_, σ_E_, G_E_, G_I _and G_s_, see Table 1) created an environmental light gradient similar to that along the depth range inhabited by sympatric cichlid species of the genus *Pundamilia *in Lake Victoria (Fig. 5d, [15]). The densities and dispersal distances used in the simulation (e.g. K, d, and N) were not unrealistic when these values are compared with the territorial size and densities in *Pundamilia *in Lake Victria. The absorbance spectrum and opsin gene expression were not exactly the same as observed in *Pundamilia *species, but our model can simulate the evolution of three photoreceptors expressing three different opsin genes to model the system of Lake Victoria cichlid fish.

We considered that speciation had occurred when two phenotypically (male color and visual sensitivity) distinct populations appeared and were maintained during the simulation period. Two populations were considered to be reproductively isolated when the number of individuals showing intermediate phenotypes between the phenotypes of the two distinct populations was less than about 5% of the population size and the two distinct populations were maintained.

## Authors' contributions

MK and OS conceived the question. MK designed and conducted the simulation programs. AS and SK contributed the design of the visual model. OS checked the validity of the simulation models based on empirical data. MK and OS wrote the paper.
